# Supplementary material for: Examine frameworks policies and strategies for effective information governance in healthcare organizations
Source: PLoS One. 2025 Jul 11;20(7):e0327496. doi: 10.1371/journal.pone.0327496 (PMC12250644; doi:10.1371/journal.pone.0327496)
Supplement: Attachment — Submitted filename: Response to Reviewers [20-01-2025].docx [file pone.0327496.s002.docx]

20 Jan. 2025

Editor-in-Chief

PLOS ONE

Dear Sir/Madam,

**Re: Responses to Reviewers and Editor’s Comments**

On behalf of my colleagues, I am submitting responses to Reviewers and Editor’s comments raised in our article “*Examine frameworks policies, and strategies for effective information governance in healthcare organizations”.*

The following areas of concern raised by the reviewers and Editor with responses (highlighted) as detailed below:

**Abstract**

1. The abstract is overly detailed, with some areas feeling cluttered. While it provides a lot of information, key results and implications are not highlighted effectively. Simplify language for better readability.

- *Thank you for your feedback. The results aspect of the abstract has been revised to read, “The level of understanding of data stewardship (2.87/4.0), familiarity with concepts of data quality (2.73/5.0) and ethical considerations and privacy (3.13/5.0) when dealing with patient health data were found to be varied among the professionals, overall rated as moderate and affirming the need for improvement. Level of knowledge and understanding on these pertinent areas were highly influenced by type of profession (p<0.001; p=0.031 and, p<0.001) respectively and been indicative that some type of health workers is much concerned about data stewardship and health information governance than others. Various methods and efforts were in place to ensure quality of data, however, hampering these attempt included factors such as poor data collection methods, lack of resources, inadequate competency and some behavioural factors…”[see lines 29-55]*

**Methodology**

2. Include a detailed explanation of how the sample was randomized (e.g., stratified random sampling?).

- *Thank you for your feedback. The detailed explanation to the sampling procedure has been included to the methodology to read, “…From a stratification based on the category of profession, a simple random sampling technique was deployed in drawing the sample from the targeted population of healthcare professionals in the various health facilities. A proportion of HCP were selected from every stratum through randomization of staff ID, giving equal opportunity to partaking in the study”.[see lines 185-189]*

3. Justify the choice of a semi-structured questionnaire by linking it to study objectives (e.g., balance of quantitative and qualitative insights).

- *Thank you for your feedback. Justification for employing semi-structured questionnaire have been included and also linked to the objectives of the study, which reads, “Closed-ended questions represented quantitative part of the study whiles opened-ended questions also intended for qualitative aspect,…Specifically, opened-ended questions solicited for qualitative data on current methods used for assessing and measuring data quality, data quality challenges, and the strategies and best practices deployed to address the challenges (all under section/objective 3 of the study). In addition, qualitative data were garnered under section 5, where HCP professionals gave measures/strategies employed by their organisation during the storage, transmission, and processing of patient data.” [see lines 192-193 & 198-203]*

4. Clarify whether written informed consent was obtained and if anonymity was guaranteed.

- *Thank you for your feedback. Clarification on the type of informed consent obtained and how optimal anonymity is ensured has been provided. [see lines 217-221]*

5. Specify how sensitive health information was handled to ensure privacy during data collection and analysis.

- *Thank you for your feedback. Information on how data privacy was ensured during collection and analysis has been specified. [see lines 220-223]*

**Results Section**

6. Some statistical tests (e.g., chi-square tests) are reported, but effect sizes and confidence intervals are not always included. This might reduce the perceived rigor. Consistently report effect sizes and confidence intervals for all statistical tests.

- *Thank you for your feedback. The results has been revised to include effect size and confidence intervals for all statistical tests. [ see table 3, 4 & 8]*

7. Clearly interpret the statistical results for non-technical readers (e.g., “This suggests a moderate relationship between profession and understanding of data stewardship”).

- *Thank you for your feedback. Interpretations to the result of all three statistical test have been revised to make them clear. [see lines 271-273 , 298-304 & 451-453 for all each interpretation respectively]*

8. Provide richer qualitative data to contextualize numerical findings. For example, quote participants describing barriers to data quality (e.g., “One participant noted, ‘Inconsistent data formats make it impossible to standardize records.’”).

- *Thank you for your feedback. Various extracts have been added to sections of the results that involved qualitative method. [see lines 321-334, 347-357 & 381-391 for the respective quality data]*

9. Simplify tables (Table 5 and Table 7) by summarizing key takeaways rather than overloading with numbers.

- *Thank you for your feedback. The content of the tables have been summarized accordingly.[see tables 5 & 7]*

10. Ensure all charts have detailed captions and legends for clarity. For instance, specify what “Measures for Privacy” include in Figure 1.

- *Thank you for your feedback. All the charts in figure 1 have been revised accordingly to include captions and legends. [see lines 493-496 & figure 1]*

**Discussion section**

11. The results indicate low levels of training on data stewardship (1.86/4.0). However, the manuscript doesn’t deeply explore why this training deficit exists or its implications.

- *Thank you for your feedback. A section of the decision has been revised to explore the possible cause of this deficit as “…This unfortunate incidence of training deficit can be associated with the fact that, management and leadership of healthcare organizations trivialize data stewardship and data quality, hence reluctant to invest into training and workshops within this arena. Consequently, the quality of clinical decision-making, management level decision-making and the general quality of care would be adversely affected because these process hinge on quality data.” [see lines 551-555]*

12. Explicitly address limitations such as:
    a. Potential biases in data collection (e.g., self-reported data).
    b. Limited generalizability to other regions or countries.
    c. Challenges in data accuracy due to reliance on cross-sectional design.

- *Thank you for your feedback. A section has been included to address all the limitations of the study. [see lines 683-684, 686-689 & 692-693]*

13. Propose specific actions to mitigate these issues in future research.

- *Thank you for your feedback. Specific mitigations strategies for future research have been added to address all the study limitations. [see lines 685-686, 689-691 & 694-696]*

14. While the manuscript mentions training and policy updates, specific recommendations for healthcare organizations, policymakers, or researchers are vague.

- *Thank you for your feedback. The recommendations section has been revised to address these concerns. [see lines 727-858]*

**Conclusion section**

15. The conclusion is understated and doesn’t sufficiently tie together the findings and implications.

- *Thank you for your feedback. The conclusion section has been revised to sufficiently capture the findings of the study. The revised conclusion reads as follows “The results of the study indicates that healthcare organizations have data governance policies established, however, the understanding of these policies and procedures was moderate. Poor investment in training on data stewardship significantly limits healthcare professionals’ level of understanding. Management and leadership poor posture on data stewardship significant leads to less attention on data stewardship, hence, low rate of training on these pertinent issues which significantly affects the quality of care delivery and decision-making processes in healthcare facilities. The level of awareness and understanding on matter of data stewardship among some profession types should be enhanced; some categories of professionals have much understanding of data stewardship than others. This skewness in knowledge affect the quality of data stewardship given that, almost all healthcare professionals handle data in one way or the other. Moreover, there are some best practices put in place to ensure data quality,…” [see lines 698-718]*
