# Supplementary material for: Examine frameworks policies and strategies for effective information governance in healthcare organizations
Source: PLoS One. 2025 Jul 11;20(7):e0327496. doi: 10.1371/journal.pone.0327496 (PMC12250644; doi:10.1371/journal.pone.0327496)
Supplement: Attachment — Submitted filename: Response to Reviewers [13-03-2025].docx [file pone.0327496.s003.docx]

13^th^ March, 2025

Editor-in-Chief

PLOS ONE

Dear Sir/Madam,

**Re: Responses to Reviewers' and Editor’s Comments**

On behalf of my colleagues, I am submitting responses to the Reviewers and Editor’s comments raised in our article “***EXAMINE FRAMEWORKS POLICIES, AND STRATEGIES FOR EFFECTIVE INFORMATION GOVERNANCE IN HEALTHCARE ORGANIZATIONS****”.*

The following areas of concern raised by the reviewers and Editor with responses (highlighted) as detailed below:

1. There are minor grammatical errors and awkward phrasing in some sections, particularly in the discussion. A language editor could help improve clarity and flow.

- *Thank you for the feedback. The grammatical errors have been rectified as recommended.*

2. While limitations are addressed, the discussion of the implications for global health settings could be expanded. For example, how applicable are these findings beyond Ghana?

- *Thank you for the feedback. Detailed applications of the findings have been added to the conclusion section of the manuscript [see line 747 – 761].*

3. Although qualitative data was included, more direct participant quotes could add depth and improve the contextualization of findings.

- *Thank you for the feedback. Additional direct participant quotes have been added to the various sections [see lines 299-300, 306-307, 315-17, 346-347& 387-388].*

4. The conclusion could more strongly tie the findings to actionable policy recommendations.

- *Thank you for the feedback. The conclusion section has been revised accordingly [see lines 719- 765].*

5. Some sections repeat earlier information, particularly between the abstract and introduction.

- *Thank you for the feedback. The abstract has been revised to do away with the repeated information [see lines 16 – 19].*

6. The description for the tables needs to be more precise for easy understanding of the reader irrespective of their professions.

- *Thank you for the feedback. The description of the various tables have been revised [see lines 255-256, 269-271, 362-363, 407-408 & 439-441]*

7. The qualitative portion of the manuscript too many back-to-back quotations without much reasoning/explanation behind using that statement as a quotation. I would also recommend using "Stated by a Doctor/Nurse/Any other healthcare professional" used at the end of each quotation referring to who might have provided this statement.

- *Thank you for the feedback. The recommended changes have been made to the quotations of the qualitative sections. [see lines 296-327, 340-358 & 375-393].*

8. The discussion part needs more supporting references with comparisons.

- *Thank you for the feedback. The discussion section has been revised with additional references and comparisons [see lines 506-585 & 623-644, also see references section for their corresponding* *bibliographies].*

9. However, I find the result section a little unclear. There's no need to provide any explanation for the results obtained (in the result section). The assumptions for the result can be later installed in the discussion section

- *Thank you for the feedback. For the purpose of understanding by a non-technical reader, the explanation and assumptions of the results are both highlighted in the results and discussion sections. This is done to enhance immediate understanding of the results by a reader [see lines 544-548, 582-585 & 666-67],*

Thank you for your reconsideration and re-evaluation prior to consideration for publication. My colleagues and I appreciate your time and effort and look forward to hearing from you.

Sincerely,

Richard Okyere Boadu (PhD)

Department of Health Information Management
School of Allied Health Sciences

College of Health and Allied Health Sciences

University of Cape Coast

Cape Coast, Ghana

[richard.boadu@ucc.edu.gh](mailto:richard.boadu@ucc.edu.gh)
